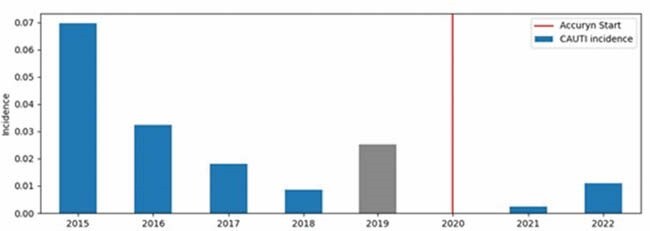# 309 Reducing the Catheter Associated Infection Rate Within a Burn Intensive Care Unit

**DOI:** 10.1093/jbcr/irad045.284

**Published:** 2023-08-29

**Authors:** David M Hill, Manu Malbrain, Vanessa Moll, Kelly P Stanton, Sai R Velamuri

**Affiliations:** Regional One Health, Memphis, Tennessee; ZNA St-Erasmus hospitals in Antwerp, Antwerp, Antwerpen; Emory University School of Medicine, Ventura, California; Potrero Medical, Austin, Texas; University of Tennessee, Memphis, Tennessee

## Abstract

**Introduction:**

Catheter-associated urinary tract infections (CAUTIs) are the most common hospital-onset healthcare-associated infections (HAI) in the United States. HAIs are key quality and safety metrics publicly reported in the acute-care space and linked to hospital reimbursement by the Centers for Medicare and Medicaid Services. CAUTIs, represent approximately 9% of all HAIs, and are associated with increased morbidity, mortality, and cost in intensive care units (ICUs). CAUTIs are thought to impact patient outcomes and healthcare costs significantly. A recent analysis concluded that CAUTIs accounted for only 0.3% of HAI costs in the United States, or approximately $28 million/year. Per patient, CAUTI costs are described in wide ranges from $876 (for inpatient costs to the hospital for additional diagnostic tests and medications) to $10,197 (for inpatient costs to Medicare for ICU patients). The objective of this study was to analyze CAUTI incidences before and after the introduction of a novel urinary catheter with an integrated active drain line clearance system for continuous urine output in a burn intensive care unit.

**Methods:**

Retrospective cohort study following the implementation of a novel urine output monitoring system with integrated drain line and urine clearance. Data from a 48-month (from January 2015-December 2018) historical control (period 1) were compared to data from a 28-month (from January 2020 to April 2022) post-implementation period (period 2). Pre- and post-implementation CAUTI event incidences were compared. Charts were reviewed to characterize the patients.

**Results:**

A total of 42 CAUTIs in 2243 patients were identified using the National Health and Safety Network (NHSN) definition during the analyzed period. There were 40 CAUTI events in period 1 and two CAUTIs in period 2. The incidence of CAUTI events pre-implementation was 0.030 (mean of 10 CAUTI events per year) compared to 0.002 (mean of 1 CAUTI event per year) post-implementation of an automatic drain line clearing UO monitoring system showing a significant reduction in CAUTI events (P< 0.01, risk ratio novel vs. gravity bladder catheter 0.071, 95% confidence interval: 0.017-0.294).

**Conclusions:**

CAUTIs were reduced in the period following the implementation of a novel urinary catheter system with an integrated active drain line and urine clearance in burn patients.

**Applicability of Research to Practice:**

Implementation of a urinary catheter system with integrated drain line and urine clearance could reduce